# Andecaliximab [Anti-matrix Metalloproteinase-9] Induction Therapy for Ulcerative Colitis: A Randomised, Double-Blind, Placebo-Controlled, Phase 2/3 Study in Patients With Moderate to Severe Disease

**DOI:** 10.1093/ecco-jcc/jjy049

**Published:** 2018-05-14

**Authors:** William J Sandborn, Bal R Bhandari, Charles Randall, Ziad H Younes, Tomasz Romanczyk, Yan Xin, Emily Wendt, Hao Chai, Matt McKevitt, Sally Zhao, John S Sundy, Satish Keshav, Silvio Danese

**Affiliations:** 1 Division of Gastroenterology, University of California San Diego, La Jolla, CA, USA; 2 Delta Research Partners, Monroe, LA, USA; 3 Gastroenterology Research America and University of Texas, San Antonio, TX, USA; 4 Gastro One, Germantown, TN, USA; 5 H-T Centrum Medyczne, Tychy, Poland; 6 Gilead Sciences, Inc., Foster City, CA, USA; 7 Translational Gastroenterology Unit, University of Oxford, Oxford, UK; 8 Inflammatory Bowel Diseases Center, Humanitas Research Hospital, Rozzano, Italy

**Keywords:** Ulcerative colitis, inflammation, matrix metalloproteinase-9

## Abstract

**Background and Aims:**

Matrix metalloproteinase-9 [MMP9] is implicated in the pathogenesis of ulcerative colitis [UC] via disruption of intestinal barrier integrity and function. A phase 2/3 combined trial was designed to examine the efficacy, safety, and pharmacokinetics of the anti-MMP9 antibody, andecaliximab [formerly GS-5745], in patients with moderately to severely active UC.

**Methods:**

Patients were randomised [1:1:1] to receive placebo, 150 mg andecaliximab every 2 weeks [Q2W], or 150 mg andecaliximab weekly [QW], via subcutaneous administration. The primary endpoint was endoscopy/bleeding/stool [EBS]-defined clinical remission [endoscopic subscore of 0 or 1, rectal bleeding subscore of 0, and at least a 1-point decrease from baseline in stool frequency to achieve a subscore of 0 or 1] at Week 8. The phase 2/3 trial met prespecified futility criteria and was terminated before completion. This study describes results from the 8-week induction phase.

**Results:**

Neither 150 mg andecaliximab Q2W or QW resulted in a significant increase vs placebo in the proportion of patients achieving EBS clinical remission at Week 8. Remission rates [95% confidence intervals] were 7.3% [2.0%–17.6%], 7.4% [2.1%–17.9%], and 1.8% [0.0%–9.6%] in the placebo, andecaliximab Q2W, and andecaliximab QW groups, respectively. Similarly, Mayo Clinic Score response, endoscopic response, and mucosal [histological] healing did not differ among groups. Rates of adverse events were comparable among andecaliximab and placebo.

**Conclusions:**

Eight weeks of induction treatment with 150 mg andecaliximab in patients with UC did not induce clinical remission or response. Andecaliximab was well tolerated and pharmacokinetic properties were consistent with those previously reported.

## 1. Introduction

Ulcerative colitis [UC], one of two main inflammatory bowel diseases [IBD], is a chronic disease of unknown aetiology, characterised by a continuous pattern of inflammation of the mucosa of the colon and rectum^1–3^ which typically consists of periods of asymptomatic remission with unpredictable recurrent episodes of bloody diarrhoea, rectal urgency, and tenesmus.^2–4^ Treatment of UC is guided by both clinical symptom severity and anatomical extent of disease.^2,3^ Patients with moderately to severely active UC that cannot be controlled with oral anti-inflammatory or immunosuppressive therapies (i.e. 5-aminosalicylate [5-ASA], corticosteroid, or thioprine [azathioprine, 6-mercaptopurine]), as well as patients dependent on corticosteroid therapy, are often treated with biological therapeutics to induce remission.^2,3,5,6^ Currently approved biological therapies target the pro-inflammatory cytokine tumour necrosis factor-alpha [TNFα] or the cell adhesion molecule α_4_β_7_ integrin, which regulates trafficking of inflammatory mediators to the intestinal epithelium,^2,5–7^ and can induce remission in some difficult-to-treat patients.^8–10^ However, the rates of sustained remission of clinical symptoms, as well as resolution of mucosal inflammation, are low,^8,9,11,12^ highlighting an unmet need for novel treatments that maintain remission in UC.

The pathogenesis of UC, although not fully understood, appears to involve the development of an enhanced immune response dependent on the presence of certain commensal enteric bacteria in genetically susceptible hosts, with exacerbations precipitated by environmental factors perturbing gastrointestinal homeostasis.^13^ Environmental factors have been associated with impaired barrier function in the intestinal epithelium and may be implicated in the development of IBD.^13–20^ Barrier disruption allows the luminal flora to translocate into the bowel wall and activate the mucosal immune response, which, in healthy individuals, is designed to prevent infection and repair the barrier.^15^ In patients with UC, several genetic factors are believed to prevent barrier repair and resolution of the acute mucosal immune response, leading to chronic intestinal inflammation.^21^ This chronic wound model of self-perpetuating, unregulated immune-mediated tissue destruction further inhibits barrier repair, allowing continued activation of mucosal immune cells by luminal flora. Increased levels of proteinases, such as the matrix metalloproteinases [MMPs], likely contribute to immune-mediated tissue destruction.^22^ Accordingly, inhibition of MMPs may inhibit cell-mediated injury and initiate wound repair in UC.^23^ No currently available therapy directly targets these unregulated proteinases involved in barrier function.

MMPs may contribute to impaired barrier function in UC through destruction of basement membranes, alterations in barrier permeability, activation and/or recruitment of pro-inflammatory cytokines, and regulation of angiogenesis.^23–26^ Specifically, the gelatinase matrix metalloproteinase-9 [MMP9] exhibits increased mucosal protein and mRNA expression, serum antigen concentrations, and activity in patients with UC compared with healthy controls.^24,26–29^ Furthermore, relative to healthy controls and patients with other types of IBD, faecal MMP9 concentrations are significantly increased in patients with UC and correlate with clinical and endoscopic activity scores.^30,31^ Targeted deletion or pharmacological inhibition of MMP9 attenuates colonic damage in experimental colitis animal models,^32–36^ indicating that MMP9-specific inhibition may represent a viable approach to the treatment of UC.

Andecaliximab [GS-5745; Gilead Sciences, Inc., Foster City, CA, USA] is a recombinant chimeric IgG4 monoclonal antibody that has been engineered to remove T-cell epitopes in an effort to reduce the risk of immunogenicity. Andecaliximab selectively binds and inhibits both latent pro-MMP9 and activated MMP9 isoforms, with negligible cross-reactivity against other MMPs including the highly homologous matrix metalloproteinase-2.^32,37^ In a recent phase 1 dose-escalation study in patients with UC, andecaliximab had good tolerability and was associated with a numerically greater percentage of clinical, endoscopic, and histological responses in patients relative to placebo over a 5-week treatment period.^38^ A phase 2/3 trial, evaluating the safety and efficacy of andecaliximab to induce and maintain clinical remission in patients with moderate to severe UC, was initiated. A planned interim futility analysis following an 8-week induction period in the first 150 patients resulted in termination of the study due to lack of efficacy. Results from the 8-week induction portion of the study are described in this report and may help increase knowledge of the pathogenesis of UC, as well as future UC drug development, with other therapeutic agents.

## 2. Materials and Methods

### 2.1. Study design and conduct

This was a combined phase 2/3 double-blind, randomised, placebo-controlled study conducted at 116 sites in 24 countries [Australia, Belgium, Bulgaria, Canada, Croatia, Czech Republic, France, Hungary, Ireland, Italy, Republic of Korea, Latvia, the Netherlands, New Zealand, Poland, Romania, Russian Federation, Slovakia, South Africa, Switzerland, Taiwan, Ukraine, the UK, and the USA]. After randomisation of 150 patients [phase 2] and before enrolment of up to 510 additional patients [phase 3], screening was halted and an interim futility analysis was conducted by an external Data Monitoring Committee. Prespecified futility criteria based on endoscopic response, defined as an endoscopic subscore of 0 or 1, determined by a blinded central reader following the 8-week induction period, were used to gate the transition from phase 2 to phase 3. Before enrolment of additional patients for phase 3 initiation, the study was terminated by the sponsor and only the phase 2 8-week induction portion of the study is reported here. A full description of the approved phase 2/3 study design, including the 52-week maintenance study and the open-label extended treatment phase, can be accessed at clinicaltrials.gov [identifier: NCT02520284].

The study was conducted in accordance with the Declaration of Helsinki, International Conference on Harmonisation guidelines, and the principles of Good Clinical Practice. The study was approved by an institutional review board/independent ethics committee at each study site before initiation, and written informed consent was obtained from all participants.

### 2.2. Patients

Males and non-pregnant, non-lactating females aged 18 to 75 years inclusive, with a documented diagnosis of UC of at least 6 months duration and with a minimum disease extent of 15 cm from the anal verge, were eligible for the study. Patients were required to meet the Mayo Clinical Score [MCS] definition of moderately to severely active UC, determined by a centrally read endoscopy score ≥2, a rectal bleeding score ≥1, a stool frequency score ≥1, and physicians’ global assessment [PGA] of 2 or 3. Endoscopies were required within 14 days of the first dose of study drug. Additionally, eligible patients must have demonstrated an inadequate response, loss of response, or intolerance to at least one oral corticosteroid, small-molecule immunomodulator [oral azathioprine or 6-mercaptopurine, or methotrexate], or biological immunomodulator [TNFα antagonists or vedolizumab] within the preceding 5 years. Patients were allowed to receive concomitant oral 5-ASA compounds or up to 30 mg daily prednisone-equivalent dose oral corticosteroids if the dose was stable for at least 2 weeks before screening, and azathioprine, 6-mercaptopurine, or methotrexate if the dose was stable for 8 weeks before screening.

Key exclusion criteria included: severe UC, defined for this study as ≥6 bloody stools daily and body temperature >38°C and/or pulse >90 beats/minute; use of rectal 5-ASA compounds or corticosteroids 2 weeks before screening; diagnosis of Crohn’s disease or indeterminate colitis; or a history of colonic or small bowel stoma, colectomy, partial colectomy, or dysplasia on biopsy. Patients with a positive stool test for *Clostridium difficile*, pathogenic *Escherichia coli*, *Salmonella*, *Shigella*, *Campylobacter*, *Yersinia*, or for ova and parasites were also excluded from the study. Patients treated with TNFα-targeted agents or vedolizumab within 8 weeks of randomisation; patients treated with investigational medicinal therapy or biologics, or non-biologic therapies other than those permitted by inclusion criteria, within 4 weeks of screening; and patients with a history of malignancy within 5 years of screening, except for those successfully treated for non-melanoma skin cancer or cervical carcinoma in situ, were also ineligible.

### 2.3. Randomisation, treatment, and dose

Eligible patients were randomised in a blinded fashion in a 1:1:1 ratio to receive subcutaneous [SC] injection of placebo weekly [QW], 150 mg andecaliximab QW, or 150 mg andecaliximab every 2 weeks [Q2W] with alternating matching placebo Q2W. Randomisation was stratified by concomitant systemic corticosteroid use and by previous history of TNFα antagonist therapy. Study visits for all patients occurred at screening and at Weeks 0 [baseline], 1, 3, 5, 7, and 8.

The study drug [150-mg andecaliximab at a concentration of 150 mg/mL] or matching placebo was supplied as a sterile, aqueous buffered solution in a single-use 1-mL prefilled syringe for SC administration. Andecaliximab or placebo was injected into either the thigh or the abdomen of each patient. Injections were administered at the research centre during study visits in the presence of the investigator or a qualified designee. Patients and/or caregivers were allowed to self-administer SC injections between study visits, provided they were deemed adequately trained by the investigator. Patients received electronic diaries for documentation of self-reported MCS components, including daily records of stool frequency and rectal bleeding.

### 2.4. Primary and secondary endpoints

The primary endpoint of the induction study was the proportion of patients achieving clinical remission, defined as an endoscopic subscore of 0 or 1, rectal bleeding subscore of 0, and at least a 1-point decrease in stool frequency from baseline to achieve a subscore of 0 or 1, at Week 8 (endoscopic/bleeding/stool [EBS] clinical remission). Week 8 assessments included a centrally reviewed flexible sigmoidoscopy/colonoscopy.

Secondary endpoints included the proportion of patients achieving MCS remission, MCS response, endoscopic remission, endoscopic response, and mucosal [histological] healing at Week 8. Additionally, the change from baseline in partial MCS was evaluated over the 8-week induction period. The MCS is composed of four subscores [stool frequency, rectal bleeding, endoscopic findings, and PGA] ranging from 0 to 3 points, with the total of subscores ranging from 0 to 12 points; the partial MCS is composed of subscores from rectal bleeding, stool frequency, and PGA. A total score ≤2 and no individual subscore >1 point was defined as MCS remission; MCS reduction of ≥3 points and at least a 30% score reduction from baseline with an accompanying decrease in rectal bleeding subscore of ≥1 point or an absolute rectal bleeding subscore of 0 or 1 was defined as MCS response. Endoscopic remission was defined as an endoscopic subscore of 0; endoscopic response was defined as an endoscopic subscore of 0 or 1. Mucosal [histological] healing was evaluated using the Geboes histological scoring system,^39^ and defined as the selection of the following combined scores: ≤3 for grade 0 [structural architectural change]; ≤1 for grade 1 [chronic inflammatory infiltrate]; ≤3 for grade 2A [lamina propria eosinophils]; and 0 for grade 2B [lamina propria neutrophils], grade 3 [neutrophils in epithelium], grade 4 [crypt destruction], and grade 5 [erosion or ulceration].

### 2.5. Pharmacokinetic assessments

Plasma samples for assessing andecaliximab drug concentrations were collected before drug administration at Weeks 1, 5, and 8. In an optional pharmacokinetic [PK] substudy, additional plasma samples were collected 3 [±1] days and 5 [±1] days after the first dose, with at least 1 day separating the two collection time points. Pharmacokinetic parameters, including the maximum observed plasma concentration [C_max_], time of the observed C_max_ [T_max_], the last observed quantifiable concentration [C_last_], time of the observed C_last_ [T_last_], and the area under the plasma concentration vs time curve from time 0 to the last quantifiable concentration [AUC_last_], were estimated based on the two samples from the PK substudy and the pre-dose sample at Week 1. Plasma concentrations of andecaliximab were determined by Biologics Development Services [Tampa, FL, USA] using a validated electrochemiluminescence [ECL] assay with a calibrated range of 25 to 3200 ng/mL and interassay precision [% coefficient of variance] of ≤14.4%. The PK parameters were estimated using Phoenix WinNonlin^®^ [Version 6.3, Pharsight Corporation, Mountain View, CA, USA] using standard non-compartmental methods.

### 2.6. Biomarker assessments

Faecal calprotectin [fCAL^®^ ELISA; EK-CAL, Bühlmann, Schönenbuch, Switzerland] and faecal lactoferrin [IBD-SCAN^®^ T5009; TECHLAB, Blacksburg, Virginia, USA] levels were assessed in stool samples collected at baseline and at the Week 8 visit. The proportion of patients achieving biomarker levels within a healthy control range were assessed at baseline and at Week 8. A cutoff of 50 µg/g for faecal calprotectin is the upper limit of normal, with levels <250 µg/g predictive of mucosal healing^40^; a cutoff of 7.24 µg/g was set for faecal lactoferrin.^41^

### 2.7. Safety

Safety assessments were conducted at all study visits and within 30 days after the last dose of study drug or early termination visit. Safety was evaluated through documentation of treatment-emergent adverse events [TEAEs], changes in vital signs, laboratory test results, and electrocardiograms [ECGs]. Severity of adverse events [AEs] was graded according to the Common Terminology Criteria for Adverse Events version 4.03.

### 2.8. Immunogenicity

Immunogenicity of andecaliximab was evaluated based upon the incidence of anti-drug antibody [ADA] formation. Serum samples for ADA analysis were collected pre-dose on Weeks 0, 1, and 5, and at Week 8. Andecaliximab ADA was detected using a validated ECL immunoassay. A multi-tiered approach that included a screening assay, a confirmatory assay for samples showing positivity in the screening assay, and a titration assay for samples showing positivity in the confirmatory assay was applied.

### 2.9. Statistical analysis

The safety analysis set included all patients who received at least one dose of study drug [andecaliximab or placebo]. The PK and immunogenicity analysis sets included patients in the safety set who had the necessary baseline and on-study measurements to provide interpretable results for the specific parameters of interest. The efficacy analysis set included all patients who were randomised at Week 0 and received at least one dose of study drug. A stratified Cochran-Mantel-Haenszel test with concomitant corticosteroid use [yes or no] and previous history of TNFα antagonist therapy [yes or no] as stratification variables was planned to compare the treatment effect between andecaliximab and placebo. Data from the phase 2 induction portion was used in the futility analysis. Due to lack of efficacy and early termination of the study by the sponsor, no formal statistical testing was conducted. Data from the phase 2 induction portion of the study were summarised by the number and percentage of patients for categorical data and by mean (standard deviation [SD]), median [min–max], or median [first quartile, third quartile] for continuous data.

## 3. Results

### 3.1. Patient disposition and demographic characteristics

**Figure 1.**
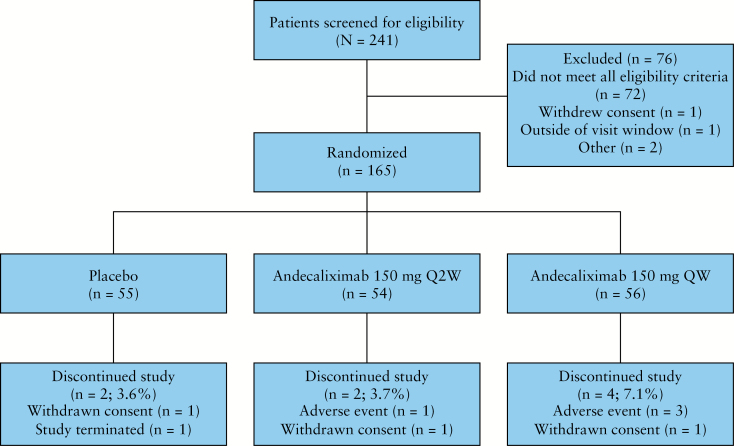
CONSORT diagram of patient disposition for 8-week induction study. Q2W, every 2 weeks; QW, weekly.

The study began September 2015; the final visit for the last enrolled patient occurred in November 2016. A total of 241 patients were screened and 165 patients were enrolled [placebo, *n* = 55; andecaliximab Q2W, *n* = 54; and andecaliximab QW, *n* = 56] [Figure 1]. Of those patients enrolled, 53 [96.4%], 52 [96.3%], and 52 [92.9%] patients in the placebo, andecaliximab Q2W, and andecaliximab QW groups, respectively, completed the 8-week induction phase. Patient demographics, disease baseline characteristics, and previous and concomitant medications were comparable across treatment groups [Table 1].

**Table 1.** Patient demographics and baseline characteristics.

Characteristic	Andecaliximab		Placebo [*n* = 55]
	150 mg Q2W [*n* = 54]	**150 mg QW** **[*n*** = **56]**	
Age, mean [SD], years	44 [14.1]	43 [13.2]	43 [12.8]
Male, *n* [%]	35 [64.8]	38 [67.9]	31 [56.4]
White, *n* [%]	48 [88.9]	48 [85.7]	45 [81.8]
Not Hispanic or Latino, *n* [%]	54 [100.0]	56 [100.0]	51 [92.7]
BMI, mean [SD], kg/m^2^	27.7 [7.6]	25.9 [6.3]	26.0 [6.0]
Duration of UC, mean [SD], years	10 [8.5]	9 [10.0]	10 [9.0]
MCS, mean [SD]	9 [1.3]	9 [1.3]	9 [1.3]^a^
Endoscopic subscore of 3, *n* [%]	34 [63.0]	39 [69.6]	37 [67.3]
Current smoker, *n* [%]	3 [5.6]	1 [1.8]	1 [1.8]
Haemoglobin, median [Q1, Q3], g/dL	13.1 [11.9, 14.1]^b^	12.5 [11.0, 13.9]	12.9 [11.4, 14.5]
Faecal calprotectin, median [Q1, Q3], µg/g	1517 [618, 2828]	1478 [406, 2523]	1513 [490, 2983]
Previous UC medications, *n* [%]			
Corticosteroids	34 [63.0]	29 [51.8]	33 [60.0]
Small-molecule immunomodulators	24 [44.4]	21 [37.5]	25 [45.5]
Biologic immunomodulators			
TNF-α antagonist	29 [53.7]	31 [55.4]	30 [54.5]
Vedolizumab	7 [13.0]	14 [25.0]	9 [16.4]
Previous UC treatment failure, *n* [%]			
TNF-α antagonist	25 [46.3]	27 [48.2]	25 [45.5]
Vedolizumab	6 [11.1]	14 [25.0]	9 [16.4]
Concomitant medications, *n* [%]			
Oral corticosteroids	20 [37.0]	22 [39.3]	17 [30.9]
Oral 5-ASA	36 [66.7]	40 [71.4]	35 [63.6]
Small-molecule immunomodulators^c^	16 [29.6]	13 [23.2]	17 [30.9]

5-ASA, 5-aminosalicylic acid; 6-MP, 6-mercaptopurine; BMI, body mass index; MCS, Mayo Clinical Score; Q1, first quartile; Q2W, every 2 weeks; Q3, third quartile; QW, weekly; SD, standard deviation; TNF-α; tumour necrosis factor-alpha; UC, ulcerative colitis.

^a^One patient did not have a baseline MCS due to site entry error.

^b^ *n* = 53.

^c^Allowed concomitant small-molecule immunomodulators were azathioprine, 6-MP, and methotrexate.

### 3.2. Efficacy endpoints

**Figure 2.**
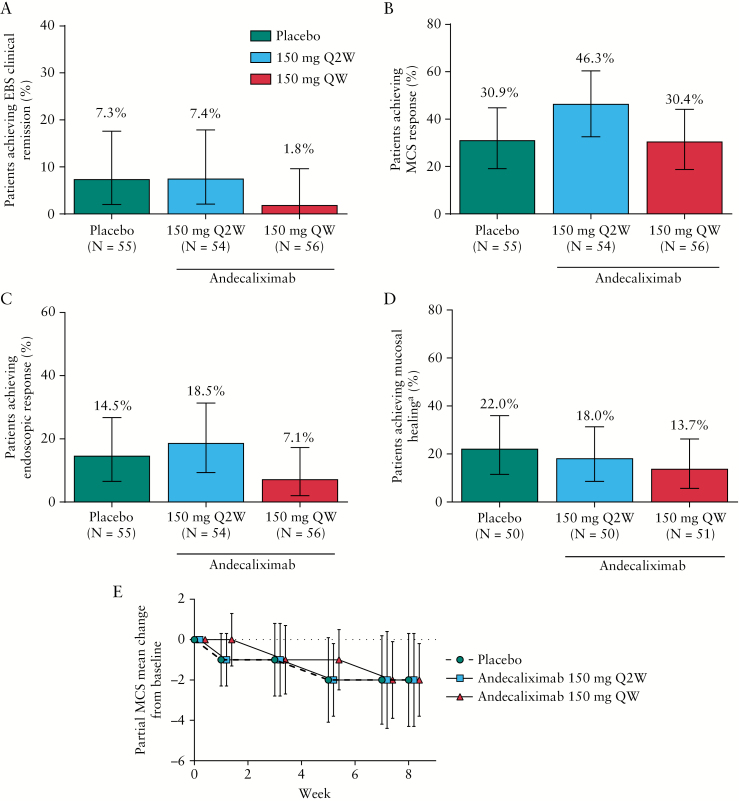
Efficacy endpoints. The proportion of patients achieving [A] EBS clinical remission, [B] MCS response, [C] endoscopic response, and [D] mucosal healing at Week 8. [E] Change from baseline in partial MCS over time. [A–D] Values indicate percentage of patients per treatment group achieving response. Error bars indicate upper and lower 95% confidence interval. [E] Data represented as mean with error bars indicating SD. EBS clinical remission defined as endoscopic subscore of 0 or 1, rectal bleeding score of 0, and ≥1-point decrease in stool frequency to achieve a subscore of 0 or 1; MCS response defined as reduction of ≥3 points and at least 30% from baseline with decrease in rectal bleeding subscore of ≥1 point or an absolute rectal bleeding subscore of 0 or 1; endoscopic response defined as endoscopic subscore of 0 or 1; mucosal healing defined as elimination of ulcers/erosion, elimination of crypt destruction, elimination of intraepithelial neutrophils, elimination of lamina propria neutrophils, and reduction in lamina propria chronic inflammatory cells to at most a mild increase. Partial MCS comprised subscores from rectal bleeding, stool frequency, and PGA. ^a^Patients with mucosal healing at baseline were excluded from the analysis. EBS, endoscopy/bleeding/stool; MCS, Mayo clinical score; PGA, physician’s global assessment; Q2W, every 2 weeks; QW, weekly; SD, standard deviation.

Both andecaliximab groups met pre-specified futility criteria and the study was terminated in September 2016, before phase 3 initiation. There was no difference observed between andecaliximab and placebo treatment groups in EBS clinical remission at Week 8, with 7.3% (95% confidence interval [CI], 2.0%–17.6%) of patients in the placebo group and 7.4% [95% CI, 2.1%–17.9%] and 1.8% [95% CI, 0.0%–9.6%] of patients in the andecaliximab Q2W and QW groups, respectively, achieving EBS clinical remission [Figure 2A]. The proportion of patients achieving MCS remission was identical to those achieving EBS clinical remission for all groups [Supplementary Table 1, available as Supplementary data at ECCO-JCC online]. There was no observed difference between either of the andecaliximab groups and placebo in the proportion of patients achieving MCS response, endoscopic remission or response, or Geboes-defined mucosal healing at Week 8 [Figure 2B–D and Supplementary Table 1]. The change from baseline in partial MCS was similar across groups over the 8-week induction period, with mean [SD] change from baseline of −2 [2.3], −2 [2.3], and −2 [1.8] in the placebo, andecaliximab Q2W, and andecaliximab QW treatment groups, respectively, at Week 8 [Figure 2E].

### 3.3. Pharmacokinetics

**Figure 3.**
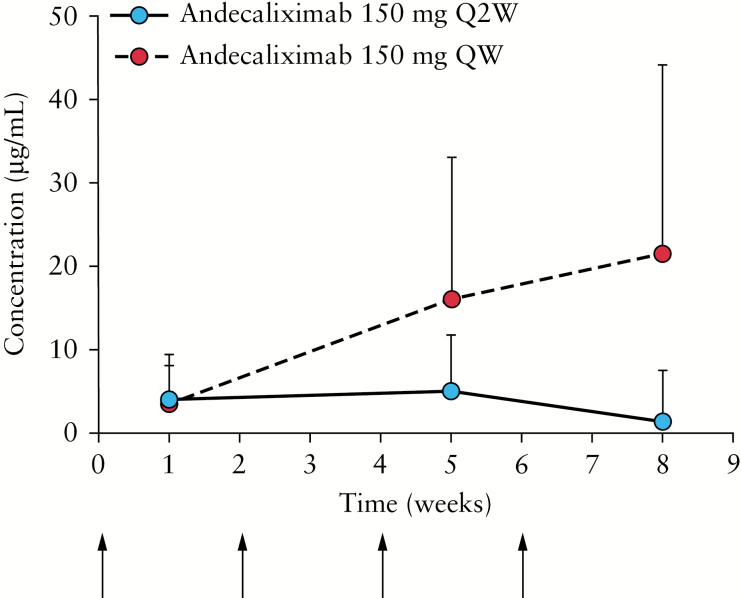
Mean plasma concentration vs time curves for andecaliximab Q2W and QW. Error bars indicate SD. Arrows indicate drug administration for Q2W dosing. All PK samples were collected before dosing on respective treatment days. For the 150 mg Q2W group, the Week 8 concentration was determined 2 weeks following the previous dose, whereas Week 1 and Week 5 concentrations were determined 1 week following the previous dose. PK, pharmacokinetic; Q2W, every 2 weeks; QW, weekly; SD, standard deviation.

The plasma concentration vs time curves for andecaliximab Q2W and QW for the 8-week induction study demonstrated accumulation of andecaliximab at Weeks 5 and 8 in the QW group, but not in the Q2W group [Figure 3]. For the PK substudy, patients receiving andecaliximab Q2W [*n* = 10] and andecaliximab QW [*n* = 12] were evaluated 3 [±1] days, 5 [±1] days, and 1 week after the first injection. As expected, the PK properties were similar between the Q2W and QW andecaliximab treatment groups [Table 2].

**Table 2.** Pharmacokinetic properties of andecaliximab.

Parameter	**Andecaliximab** **150 mg Q2W** **[*n*** = **10]**	**Andecaliximab** **150 mg QW** **[*n*** = **12]**
AUC_last_, day*µg/mL	62.0 [51.5]	63.4 [44.1]
C_max_, µg/mL	13.6 [10.1]	14.9 [10.3]
T_max_, day, median	3.00 [2.00, 4.00]	3.00 [2.50, 4.50]
C_last_, µg/mL	5.6 [6.75]	3.8 [3.59]
T_last_, day	7.00 [7.00, 7.00]	7.00 [7.00, 8.00]

All data are presented as mean [SD] except for T_max_ and T_last_, which are presented as median [Q1, Q3].

AUC_last_, area under the plasma concentration vs time curve from time 0 to the last quantifiable concentration; C_last_, the last observed quantifiable concentration; C_max_, maximum observed plasma concentration; Q1, first quartile; Q2W, every 2 weeks; Q3, third quartile; QW, weekly; SD, standard deviation; T_last_, time of the observed C_last_; T_max_, time of the observed C_max_.

### 3.4. Biomarkers

For each treatment group, the proportions of patients achieving disease biomarker concentrations within a healthy control range or associated with mucosal healing were evaluated at baseline and Week 8. The proportion of patients achieving faecal lactoferrin <7.24 µg/g was relatively unchanged at Week 8 compared with baseline in all treatment groups [Table 3]. Following andecaliximab Q2W and QW treatment, the proportion of patients achieving faecal calprotectin levels ≤50 µg/g was relatively unchanged at Week 8 compared with baseline, whereas a higher percentage of patients achieved faecal calprotectin levels ≤250 µg/g at Week 8 compared with baseline. However, a similar trend was also observed in placebo-treated patients [Table 3].

**Table 3.** Proportion of patients below clinically meaningful levels in disease biomarkers at baseline and Week 8.

Biomarker, *n*/*N* [%]	Andecaliximab 150 mg Q2W		Andecaliximab 150 mg QW		Placebo QW	
	Baseline	**Week 8**	**Baseline**	**Week 8**	**Baseline**	**Week 8**
Faecal calprotectin						
≤50.0 µg/g	0/50 [0]	4/47 [8.5]	2/48 [4.2]	4/48 [8.3]	2/50 [4.0]	3/46 [6.5]
≤250 µg/g	3/50 [6.0]	18/47 [38.3]	6/48 [12.5]	17/48 [35.4]	6/50 [12.0]	15/46 [32.6]
Faecal lactoferrin						
<7.24 µg/g	2/50 [4.0]	8/45 [17.8]	5/50 [10.0]	9/49 [18.4]	3/50 [6.0]	6/48 [12.5]

Q2W, every 2 weeks; QW, weekly.

### 3.5. Immunogenicity

There was no positive ADA response in the placebo group at any study visit. At baseline and following one injection of andecaliximab [Week 1], no positive ADA results were observed; positive ADA responses were first detected at Week 5. During the course of the 8-week induction study, 13/52 [25%] of the tested patients in the andecaliximab Q2W and 9/54 [16.7%] of the tested patients in the andecaliximab QW groups exhibited treatment-induced positive ADA response.

### 3.6. Safety

During the induction study, 29 [53.7%] patients in the andecaliximab Q2W group, 33 [58.9%] patients in the andecaliximab QW group, and 33 [60.0%] patients in the placebo group experienced at least one TEAE. No serious AEs [SAEs] were observed in the andecaliximab Q2W group, whereas two [3.6%] patients in the andecaliximab QW [anaemia and angina pectoris] and one [1.8%] patient in the placebo group [anal abscess] experienced SAEs. One [1.9%] patient receiving andecaliximab Q2W, three [5.4%] patients receiving andecaliximab QW, and one [1.8%] patient receiving placebo experienced TEAEs, resulting in premature study discontinuation. Anaemia and headache were the most common TEAEs in andecaliximab-treated patients, and abdominal pain was the most common TEAE in placebo-treated patients [Table 4]. Marked laboratory abnormalities were experienced by three [5.6%] patients in the andecaliximab Q2W group [lymphocyte count decreased, creatinine kinase increased, and hypophosphataemia]; five [8.9%] patients in the andecaliximab QW group (lymphocyte count decreased, hypocalcaemia, creatinine kinase increased, hyperglycaemia [*n* = 2], and hypophosphataemia); and five [9.1%] patients in the placebo group (lymphocyte count decreased, creatinine kinase increased, hyperglycaemia, and hypophosphataemia [*n* = 2]) over the 8-week induction phase. No clinically significant changes from baseline in ECGs occurred.

**Table 4.** Treatment-emergent adverse events occurring in ≥5% of patients in any treatment group.

Preferred term	Andecaliximab		
	150 mg Q2W [*n* = 54]	**150 mg QW** **[*n*** = **56]**	**Placebo** **[*n*** = **55]**
Anaemia	4 [7.4]	5 [8.9]	2 [3.6]
Abdominal pain	1 [1.9]	2 [3.6]	5 [9.1]
Nausea	2 [3.7]	3 [5.4]	3 [5.5]
Arthralgia	1 [1.9]	3 [5.4]	3 [5.5]
Headache	2 [3.7]	4 [7.1]	1 [1.8]
Back pain	1 [1.9]	3 [5.4]	2 [3.6]
Colitis ulcerative	2 [3.7]	3 [5.4]	1 [1.8]
Pyrexia	1 [1.9]	4 [7.1]	1 [1.8]
Cough	1 [1.9]	3 [5.4]	1 [1.8]
Fatigue	1 [1.9]	1 [1.8]	3 [5.5]
Hyperglycaemia	0	4 [7.1]	1 [1.8]
Injection site bruising	1 [1.9]	4 [7.1]	0
Sinusitis	0	3 [5.4]	0

All data are presented as a [%]. Adverse events were defined according to MedDRA v19.1. Multiple adverse events were counted only once per patient for each preferred term.

Q2W, every 2 weeks; QW, weekly.

## 4. Discussion

The phase 2/3 combined trial of andecaliximab for the treatment of moderately to severely active UC was terminated based on interim futility analysis following the 8-week induction period of the trial. Rates of EBS clinical remission, MCS clinical remission and response, endoscopic remission and response, and mucosal healing were unchanged by administration of andecaliximab relative to placebo treatment. Consistent with lack of treatment effect of andecaliximab, changes in concentrations of disease biomarkers, including faecal calprotectin and faecal lactoferrin, were similar relative to placebo.

Results from a recent phase 1 dose-escalation study of andecaliximab in patients with moderately to severely active UC suggested greater clinical response, clinical remission, and endoscopic response rates relative to placebo in patients receiving 150 mg SC QW andecaliximab (or equivalent intravenous [IV] doses) for 5 weeks.^38^ Furthermore, both human and animal studies demonstrate that MMP9 may contribute to UC disease pathogenesis. Similar to UC in human disease, in animal models of dextran sodium sulphate [DSS]-induced colitis, MMP9 expression is greatly increased in neutrophilic infiltrates and co-localises to regions of epithelial and endothelial basement membrane destruction.^26,32,33^ Several studies have demonstrated that genetic ablation or pharmacological inhibition of MMP9 attenuates disease severity and tissue damage in the DSS UC model.^32–36^ As such, the fact that specific inhibition of MMP9 with andecaliximab was ineffective in reducing UC disease severity in this study was surprising. It should be noted, however, that a more recently published non-clinical study of multiple animal models of IBD failed to demonstrate a beneficial effect of peptide inhibitors of MMP9, and MMP9 knockout mice did not differ from wild-type mice in disease severity of DSS- or 2,4,6-trinitrobenzenesulphonic acid-induced colitis.^42^ Furthermore, a recent phase 2 trial of andecaliximab in Crohn’s disease yielded similar negative results [see companion paper].

In phase 1 testing, administration of andecaliximab [150 mg SC weekly] had comparable clinical benefit to 0.3–3 mg/kg administered IV Q2W, and was selected in the phase 2/3 study based on convenience, as patients can self-administer the study drug using prefilled syringes, and as there is reduced risk of injection-site or systemic reactions compared with IV dosing. However, as only 10 patients received SC 150 mg andecaliximab in the phase 1 study, it is possible this small patient cohort did not accurately reflect the general UC population and patient numbers may have been too small to draw conclusions regarding efficacy.

Selection of an 8-week induction period is consistent with similar UC trials and is accepted within the field as an appropriate period of time to evaluate clinical response and endoscopic improvement. However, it is possible that treatments that target barrier function take longer to impact disease resolution. Endoscopies were centrally read in a blinded manner to minimise potential random variation between placebo and andecaliximab treatment groups. Here, the rate of MCS remission in placebo-treated patients [7.3%] is comparable to remission rates in placebo-treated patients previously reported in induction treatment trials in UC without central reading.^8–10,12,43^ However, in the phase 1 study in which endoscopies were also performed by a central reader, 0% of placebo-treated patients experienced clinical remission.^38^ This exceptionally low placebo remission rate, which was not repeated in the phase 2/3 trial, may have contributed to the difference seen between placebo- and andecaliximab-treated patients in the phase 1 study.

Differences in the study population between the phase 1 study and this phase 2/3 study may have contributed to the discrepancy in findings. For example, for the phase 2/3 trial, eligibility criteria required that patients had previously experienced inadequate response to at least one of corticosteroids, azathioprine, 6-mercaptopurine, methotrexate, or TNF-antagonists, resulting in a higher percentage of total patients previously exposed to anti-TNFα agents than in the phase 1 study [55% vs 20%], in which this eligibility restriction was not applied. Therefore, the phase 2/3 study patients may have comprised a more difficult-to-treat population compared with the phase 1 study population.

The andecaliximab PK profile was in accordance with previous studies,^38,44^ suggesting that unexpected PK properties did not account for the observed lack of clinical efficacy. Treatment-induced positive ADA response occurred in 20.8% of andecaliximab-treated patients during the 8-week induction period, with a numerically higher proportion of ADA-positive patients in Q2W compared with QW dosing groups [25.0% vs 16.7%]. Due to differences in ADA assays [e.g. methodology and assay sensitivity and specificity], it is difficult to compare the immunogenicity of therapeutic proteins.^45^ Regardless, the positive ADA did not seem to affect andecaliximab PK in this study. Validated measures for determination of MMP9 coverage in stool or colonic tissue are unavailable; therefore, a potential limitation to this study is that it cannot be determined whether the selected dosing regimens resulted in sufficient andecaliximab to adequately bind and neutralise the elevated level of MMP9 present in diseased tissue.

No deaths occurred during the study. There were three SAEs [*n* = 2 in andecaliximab-treated patients] and five TEAEs [*n* = 4 in andecaliximab-treated patients], leading to premature study discontinuation. Although pan-MMP inhibitors have demonstrated significant side effects, including the development of musculoskeletal toxicity in previous clinical trials,^46,47^ no evidence of musculoskeletal syndrome occurred in this study.

Andecaliximab was not effective in producing a clinical or endoscopic response as induction therapy in patients with moderate to severe UC. All doses of andecaliximab were well tolerated, and AEs associated with older pan-MMP inhibitors were not observed in this study. Although andecaliximab failed to improve barrier function based on endoscopic and histological assessments in this study design, it remains to be seen whether improved barrier function will offer protection in IBD.

## Funding

This work was supported by the sponsor, Gilead Sciences, Inc. The study sponsor was responsible for study design, execution, data analysis, and approved submission of the final manuscript.

## Conflict of Interest

WJS reports consulting fees, research grants or speaker fees from Abbott, ActoGeniX NV, AGI Therapeutics, Inc., Alba Therapeutics Corp., Albireo, Alfa Wasserman, Amgen, Inc., AM-Pharma BV, Anaphore, Astellas Pharma, Inc., Athersys, Inc., Atlantic Healthcare Ltd., Aptalis, BioBalance Corp., Boehringer Ingelheim, Bristol-Myers Squibb, Celgene Corp., Celek Pharmaceuticals, Cellerix SL, Cerimon Pharmaceuticals, Inc., ChemoCentryx, Inc., CoMentis, Inc., Cosmo Technologies Ltd., Coronado Biosciences, Inc., Cytokine Pharmasciences, Inc., Eagle Pharmaceuticals, Inc., EnGene, Inc., Eli Lilly, Enteromedics, Exagen Diagnostics, Inc., Ferring Pharmaceuticals, Inc., Flexio Therapeutics, Inc., Funxional Therapeutics Ltd., Genentech, Inc., Genzyme Corp., Gilead Sciences, Inc., Given Imaging, GSK, Human Genome Sciences, Ironwood Pharmaceuticals, Janssen, KaloBios Pharmaceuticals, Inc., Lexicon Pharmaceuticals, Inc., Lycera Corp., Meda Pharmaceuticals, Inc., Merck Research Laboratories, Merck Serono, Millennium Pharmaceuticals, Inc., Nisshin Kyorin Pharmaceuticals, Novartis, Novo Nordisk, NPS Pharmaceuticals, Inc., Optimer Pharmaceuticals, Inc., Orexigen Therapeutics, Inc., PDL Biopharma, Pfizer, Inc., Procter & Gamble, Prometheus Laboratories, Inc., ProtAb Ltd., Purgenesis Technologies, Inc., Relypsa, Inc., Roche, Salient Pharmaceuticals, Inc., Salix Pharmaceuticals, Inc., Santarus, Inc., Schering-Plough Corp., Shire Pharmaceuticals, Sigmoid Pharma Ltd., Sirtris Pharmaceuticals, Inc., SLA Pharma UK Ltd., Targacept, Inc., Teva Pharmaceuticals, Therakos, Inc., Tillotts Pharma AG, TxCell SA, UCB Pharma, Viamet Pharmaceuticals, Inc., Vascular Biogenics Ltd., Warner Chilcott UK Ltd., Wyeth, and University of Western Ontario [owner of Robarts Clinical Trials]; and holds stocks/stock options in Enteromedics. BRB has no disclosures to report. CR has served as a speaker, consultant, or advisory board member for Boston Scientific, Forest Labs, Ironwood Pharmaceuticals, Janssen, Prometheus, Salix Pharmaceuticals, Santarus Pharmaceuticals, Takeda, and UCB. ZY has received grants or speaker fees from AbbVie, Arena Pharmaceuticals, Celgene, Eli Lilly, Genentech, Gilead Sciences, Inc., Janssen, Pfizer, and Takeda. TR has no disclosures to report. YX, EW, HC, MM, SZ, and JS are employees of and hold stock in Gilead Sciences, Inc. SK has received personal fees or grants from AbbVie, Inc., Allergan, ChemoCentryx, Inc., GlaxoSmithKline, PLC, Mitsubishi Tanabe Pharma, MSD, Pharmacosmos, Pfizer, Inc., Roche, Takeda, and Vifor Pharma. SD has served as a speaker, consultant, and/or advisory board member for AbbVie, Inc., Alfa Wassermann, Allergan, Biogen, Boehringer Ingelheim, Celgene Corp., Celltrion, Inc., Ferring Pharmaceuticals, Inc., Gilead Sciences, Inc., Hospira, Johnson & Johnson, Merck, MSD, Mundipharma, Pfizer, Inc., Sandoz, Takeda, TiGenix, UCB Pharma, and Vifor Pharma.

## Author Contributions

All authors had full access to all of the study data and had the final responsibility for the decision to submit the manuscript for publication.

## Supplementary Data

Supplementary data are available at *ECCO-JCC* online.

Supplementary Material

## Notes

Conference presentation: this work was presented at UEG Week 2017, Barcelona, Spain.
