# Supplementary material for: Andecaliximab [Anti-matrix Metalloproteinase-9] Induction Therapy for Ulcerative Colitis: A Randomised, Double-Blind, Placebo-Controlled, Phase 2/3 Study in Patients With Moderate to Severe Disease
Source: J Crohns Colitis. 2018 May 14;12(9):1021–9. doi: 10.1093/ecco-jcc/jjy049 (PMC6113706; doi:10.1093/ecco-jcc/jjy049)
Supplement: Supplementary Material [file jjy049_suppl_supplementary-material.docx]

**Supplementary Table 1.** Secondary efficacy endpoints

|  | **Andecaliximab** | |  |
| --- | --- | --- | --- |
| **Parameter** | **150 mg Q2W**  **(n = 54)** | **150 mg QW**  **(n = 56)** | **Placebo**  **(n = 55)** |
| MCS remission | 7.4 (2.1−17.9) | 1.8 (0.0−9.6) | 7.3 (2.0−17.6) |
| MCS response | 46.3 (32.6−60.4) | 30.4 (18.8−44.1) | 30.9 (19.1−44.8) |
| Endoscopic remission | 3.7 (0.5−12.7) | 0 (0.0−6.4) | 5.5 (1.1−15.1) |
| Endoscopic response | 18.5 (9.3−31.4) | 7.1 (2.0−17.3) | 14.5 (6.5−26.7) |
| Mucosal healing | 18.0 (8.6−31.4) | 13.7 (5.7−26.3) | 22.0 (11.5−36.0) |

All data are presented as the percentage of subjects achieving endpoint (95% confidence interval). MCS remission defined as total score ≤2 points and no individual subscore >1 point; MCS response defined as reduction of ≥3 points and at least 30% from baseline with decrease in rectal bleeding subscore of ≥1 point or an absolution rectal bleeding subscore of 0 or 1; endoscopic response defined as endoscopic subscore of 0 or 1; mucosal healing defined by Geboes histologic scoring system as ≤3 for grade 0 (structural architectural change), ≤1 for grade 1 (chronic inflammatory infiltrate), ≤3 for grade 2A (lamina propria eosinophils, and 0 for grade 2B (lamina propria neutrophils), grade 3 (neutrophils in the epithileium), grade 4 (crypt destruction), and grade 5 (erosion or ulceration). MCS, Mayo clinical score; Q2W, every 2 weeks; QW, weekly.
